# GDP-Mannose Pyrophosphorylase: A Biologically Validated Target for Drug Development Against Leishmaniasis

**DOI:** 10.3389/fcimb.2019.00186

**Published:** 2019-05-31

**Authors:** Sébastien Pomel, Wei Mao, Tâp Ha-Duong, Christian Cavé, Philippe M. Loiseau

**Affiliations:** UMR 8076 CNRS BioCIS, Université Paris-Sud, Université Paris-Saclay, Châtenay-Malabry, France

**Keywords:** GDP-mannose pyrophosphorylase, *Leishmania*, therapeutic target, inhibitors, drug development

## Abstract

Leishmaniases are neglected tropical diseases that threaten about 350 million people in 98 countries around the world. In order to find new antileishmanial drugs, an original approach consists in reducing the pathogenic effect of the parasite by impairing the glycoconjugate biosynthesis, necessary for parasite recognition and internalization by the macrophage. Some proteins appear to be critical in this way, and one of them, the GDP-Mannose Pyrophosphorylase (GDP-MP), is an attractive target for the design of specific inhibitors as it is essential for *Leishmania* survival and it presents significant differences with the host counterpart. Two GDP-MP inhibitors, compounds **A** and **B**, have been identified in two distinct studies by high throughput screening and by a rational approach based on molecular modeling, respectively. Compound **B** was found to be the most promising as it exhibited specific competitive inhibition of leishmanial GDP-MP and antileishmanial activities at the micromolar range with interesting selectivity indexes, as opposed to compound **A**. Therefore, compound **B** can be used as a pharmacological tool for the development of new specific antileishmanial drugs.

## Introduction

Leishmaniases are vector-borne neglected tropical diseases caused by a protozoan parasite from the genus *Leishmania* and transmitted by hematophagous female phlebotomine sandflies. During its life cycle, the parasite alternates from a promastigote motile form within the phlebotome to an intracellular amastigote form in mammalian host macrophages. Leishmaniases can be classified in three main groups according to their clinical manifestations: cutaneous, which is the most common form, muco-cutaneous leading to nasal and oropharyngeal lesions and marked disfigurements, and visceral, the most severe form, always fatal in the absence of adequate treatment. These clinical manifestations can be provoked by several *Leishmania* species: for instance, *L. major* or *L. mexicana* will give rise to cutaneous leishmaniasis, and *L. donovani* or *L. infantum* visceral leishmaniasis. Only few drugs are currently available for the treatment of leishmaniases. Antimonials, which have been historically used since 1920s, generate a strong toxicity at cardiac, renal, and hepatic levels and select drug resistance. The other classical drugs, namely oral miltefosine, injectable liposomal amphotericin B, and paromomycin, display some deleterious effects and now represent a potential threat of drug resistance as well (Croft et al., 2006; Sundar and Singh, 2016; Ponte-Sucre et al., 2017). The development of new antileishmanial treatments is thus crucial in this context.

In order to overcome the limitations of the existing treatments, rational approaches have been used to develop new specific therapies for leishmaniases (Zulfiqar et al., 2017). Among the different strategies elaborated, the identification of new targets that are essential for parasite viability or virulence is an attractive approach for the development of specific antileishmanial compounds (Jiang et al., 1999; Burchmore et al., 2003; Jain and Jain, 2018). Indeed, these essential targets can be exploited by chemical screening in order to characterize inhibitor scaffolds whose specificities are optimized by pharmacomodulations, based on target three-dimensional structures. In this way, targets from *Leishmania* energy metabolism (i.e., glycolysis, folate or redox metabolism) were first intensively studied (Aronov et al., 1999; Chowdhury et al., 1999; Verlinde et al., 2001; Olin-Sandoval et al., 2010; Colotti et al., 2013; Leroux and Krauth-Siegel, 2016). Other biochemical pathways were also investigated, but the characterized inhibitors met some limitations such as parasite specificity, inhibitors synthesis cost and lack of *in vivo* activity (Croft and Coombs, 2003).

## Targeting Membrane Glycoconjugate Metabolism

There are two main ways to impair parasite development within the host, considering proteins expressed in the amastigote form as therapeutic targets. The first one relies on targeting some biochemical pathways leading to an unbalanced metabolism, toxic for the parasite. Many proteins have been considered for this purpose (Aronov et al., 1999; Chowdhury et al., 1999; Verlinde et al., 2001; Olin-Sandoval et al., 2010). The second one considers that a relevant Achille's heel consists in avoiding macrophage-parasite interactions (Descoteaux et al., 1995; Descoteaux and Turco, 1999; Podinovskaia and Descoteaux, 2015; Lamotte et al., 2017). As host-*Leishmania* interactions mainly rely on glycoconjugate recognition, an inhibition of glycoconjugate biosynthesis could affect this molecular recognition, and therefore reduce parasite burden. Furthermore, as the glycosylation is a crucial pathway for macrophage infection (Descoteaux et al., 1995; Descoteaux and Turco, 1999; Pomel and Loiseau, 2013; Podinovskaia and Descoteaux, 2015), we hypothesize that an alteration of glycoconjugate structures would not easily select drug resistance.

**Figure 1:**
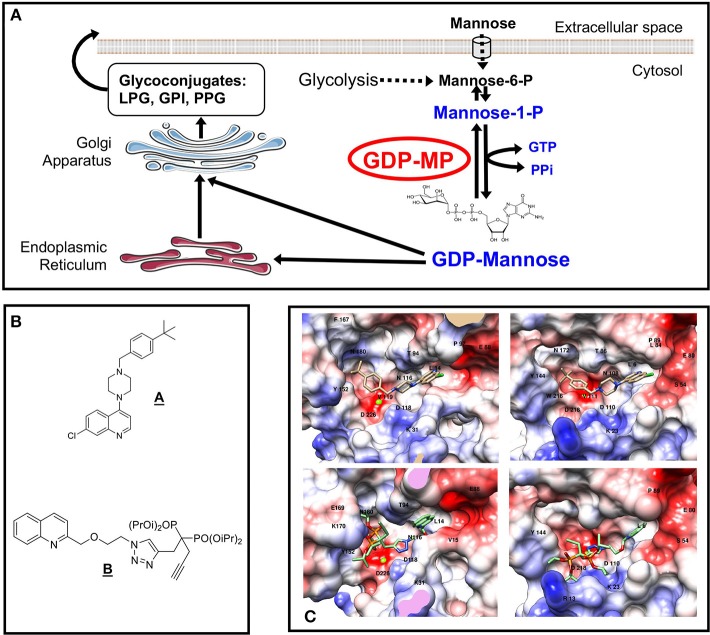
Mannose activation pathways and GDP-MP inhibitors. **(A)** Mannose activation pathways and glycoconjugate biosynthesis in *Leishmania*. The GDP-MP substrates and products are indicated in blue. The GDP-MP is circled in red. **(B)** Chemical structures of compounds **A,B**. **(C)** Docking analyses of compound **A** (top) and **B** (bottom) in LdGDP-MP (left) and hGDP-MP (right) catalytic sites. The protein surface is colored as a function of the charge density: red, white, and blue colors indicating negative, neutral, and positive area, respectively. Magnesium ion is represented by a green sphere. The amino acids that make contact with compound **A,B** in the catalytic sites are indicated in their one-letter code and number in the sequence (Adapted from Daligaux et al., 2016a; Mao et al., 2017).

Mannose-containing glycoconjugates represent a large proportion of the carbohydrates addressed at the surface of a eukaryotic cell and are involved in many biological processes such as intercellular recognition, adhesion or signaling (Varki, 2007; Colley et al., 2017). In *Leishmania*, a wide range of unusual mannose-containing glycoconjugates [e.g., GlycosylPhosphatidylInositol (GPI) anchors, LipoPhosphoGlycans (LPG), ProteoPhosphoGlycans (PPG) or GlycosylInositolPhosphoLipids (GIPLs)] are synthesized and are essential for parasite virulence (Descoteaux and Turco, 1999; Pomel and Loiseau, 2013). The biosynthesis of these glycoconjugates requires initially the conversion of mannose into GDP-mannose. The mannose moiety of this nucleotide sugar is then transferred into nascent glycoconjugates to allow mannosylation reaction. In eukaryotic cells, mannose can either be imported via membrane transporters or be generated from the reaction catalyzed by the PhosphoMannose Isomerase (PMI) on fructose-6-phosphate originating from glycolysis to produce mannose-6-phosphate (Figure 1A). In the mannosylation pathway, the PhosphoMannoMutase (PMM) converts mannose-6-phosphate in mannose-1-phosphate (Figure 1). The activated form of mannose, GDP-mannose, is then produced by the action of the GDP-Mannose Pyrophosphorylase (GDP-MP) according to the following reversible enzymatic reaction (Ning and Elbein, 2000):

Mannose − 1 − P + GTP ⇄ GDP − Mannose + PPi

The GDP-MP is a ubiquitous enzyme found in bacteria, fungi, plants, and animals and belonging to the family of nucleotidyl-transferases. In mammalian organisms, GDP-MP was mainly studied in swine (Szumilo et al., 1993; Ning and Elbein, 2000). The swine native enzyme is a complex of about 450 kDa with two distinct subunits: α (43 kDa) and β (37 kDa). In pig, as well as in human, the β subunit displays the enzymatic activity, while the α subunit would have a regulatory function (Szumilo et al., 1993; Ning and Elbein, 2000; Carss et al., 2013; Koehler et al., 2013). In human, α and β subunits share 32% identity. Mutations in the genes coding for α or β subunits in human lead to glycosylation disorders characterized notably by neurological deficits and muscular dystrophies (Carss et al., 2013; Koehler et al., 2013). Two β isoforms, named β1 and β2, have been characterized in the human genome, displaying 90 and 97% identity with the porcine β subunit, respectively. The human β2 isoform is strongly expressed in a wide range of tissues, in opposition to β1 which is only weakly expressed, especially in liver, heart, and kidney (Carss et al., 2013). Additionally, the β2 isoform shows a better homology with *Leishmania mexicana* GDP-MP, compared to β1 (49% for β2 vs. 46% for β1). In bacteria, GDP-MP are mostly dimeric, either mono- or bifunctional, the latter displaying both GDP-MP and PMI activities in separate domains of an individual enzyme (Shinabarger et al., 1991; May et al., 1994; Ning and Elbein, 1999; Wu et al., 2002; Asencion Diez et al., 2010; Pelissier et al., 2010; Akutsu et al., 2015). Unlike in other organisms, leishmanial GDP-MP has been shown to assemble as a hexamer of 240 kDa in several *Leishmania* species (Davis et al., 2004; Mao et al., 2017). As this hexamer can dissociate at low ionic strength conditions and at low protein concentration, a mixture of the three forms may be present in the reaction medium *in vitro*.

Both human and leishmanial GDP-MP have been reported to display a high substrate specificity (Mao et al., 2017), in agreement with previous studies performed in bacterial, trypanosomal, and swine GDP-MP (Ning and Elbein, 2000; Denton et al., 2010; Pelissier et al., 2010). The investigation of the mechanism of reaction has shown a sequential ordered mechanism in most bacterial GDP-MP like in some other nucleotidyl-transferases, with GTP fixation prior to mannose-1-phosphate (Barton et al., 2001; Zuccotti et al., 2001; Asencion Diez et al., 2010; Pelissier et al., 2010; Boehlein et al., 2013). However, leishmanial and human GDP-MP have been characterized by a sequential random mechanism (Mao et al., 2017), in which the substrate binding order is not defined, in agreement with a mammalian nucleotidyl-transferase (Persat et al., 1983), suggesting that the GDP-MP mechanism of reaction differs from bacteria to *Leishmania* and human.

A knockout of the gene encoding for GDP-MP in *L. mexicana* lead to an absence of development in the macrophage *in vitro* and to an absence of parasite persistence *in vivo* (Garami and Ilg, 2001; Stewart et al., 2005). These results show that GDP-MP is critical for amastigote survival and is therefore an interesting drug therapeutic target to be exploited for antileishmanial drug development. Likewise, GDP-MP has been described to be essential for cell integrity and survival in other microorganisms such as *Trypanosoma brucei, Aspergillus fumigatus*, or *Candida albicans* showing the biological validation as a potential therapeutic target of this enzyme in several kinetoplastids and fungi (Warit et al., 2000; Jiang et al., 2008; Denton et al., 2010). Additionally, a High-Throughput Screening (HTS) assay, allowed the selection of leishmanial GDP-MP inhibitors (Lackovic et al., 2010). From this study, the most potent inhibitor identified was a piperazinyl quinoline derivative (compound **A**; Figure 1B) demonstrating an *in vitro* activity on *L. major* GDP-MP and on intracellular parasite proliferation with IC_50_ values at 0.58 and 21.9 μM, respectively.

## Computational and Target-Based Drug Design

A molecular model of the GDP-MP quaternary structure has been generated in *L. mexicana*, confirming the hexameric structure of the enzyme (Perugini et al., 2005). Based on this model, GDP-MP hexamers would be assembled by a contact between trimer structures in a head-to-head manner involving only the N-terminal end of the protein. These results are however in opposition to crystallography studies of other GDP-MP or nucleotidyl-transferases, showing a tail-to-tail arrangement of the C-terminal β-helices in their quaternary structures (Cupp-Vickery et al., 2005; Jin et al., 2005; Pelissier et al., 2010; Führing et al., 2015).

As no GDP-MP crystal could be obtained in *Leishmania*, molecular models of *L. infantum* and *L. donovani* GDP-MP were generated using distinct sequence alignment strategies and were compared with the human counterpart (Pomel et al., 2012; Daligaux et al., 2016a). Both analyses showed a structural conservation of a consensus sequence GXGXRX_*n*_K in leishmanial and human GDP-MP corresponding to a pyrophosphorylase signature motif, as well as the F(V)EKP sequence previously described to be part of the GDP-MP active site (Sousa et al., 2008). Interestingly, several specific residues have been identified in the catalytic site of both *L. infantum* and *L. donovani* GDP-MP compared to the human counterpart (Pomel et al., 2012; Daligaux et al., 2016a). Moreover, GDP-MP sequences share more than 85% of identity in the *Leishmania* genus. Therefore, the differences identified between the leishmanial and human catalytic sites could potentially be exploited to design specific antileishmanial agents.

The GDP-mannose, as a substrate or a product of the GDP-MP, has been selected as the basis for inhibitor design because of its steric volume presenting the maximum of interactions within the enzyme catalytic pocket (Mao et al., 2017). In this work, the chemical approach to design leishmanial GDP-MP inhibitors relied on the pharmacomodulation of the GDP-mannose from the analysis of enzyme molecular models, by substituting for example the mannose moiety by a phenyl group, the pyrophosphate by a triazole or a phosphonate, the ribose by an ether oxide group or a deoxyribose and the guanine by different heterocycles such as purine analogs or quinolines, especially two-substituted quinolines which have been previously described to display promising *in vitro* and *in vivo* antileishmanial activities (Fournet et al., 1993, 1994, 1996; Nakayama et al., 2005, 2007; Campos-Vieira et al., 2008; Loiseau et al., 2011). Therefore, the presence of two-substituted quinolines in these compounds designed could potentiate their antileishmanial activities through GDP-MP inhibition.

## Cell-Free *in vitro* and *in silico* Evaluation of Compounds on Purified GDP-MPs

From the analysis of GDP-MP structural models, a library of 100 compounds was designed and synthesized (Daligaux et al., 2016b; Mao et al., 2017). These compounds were evaluated on recombinant GDP-MP purified from *L. donovani* (*Ld*GDP-MP), *L. mexicana* (*Lm*GDP-MP), and human (*h*GDP-MP). In this work, the *h*GDP-MP corresponded to the β2 subunit displaying the enzyme activity and showing the highest homology with leishmanial GDP-MP (see above). This evaluation allowed to identify compound **B**, a quinoline derivative substituted in position 2 with a methoxy-ethyl-triazol-butyn-diisopropylphosphonate group (Figure 1B), as a specific competitive inhibitor of *Ld*GDP-MP with a *K*_*i*_ at 7 μM. In comparison, compound **A**, previously identified from a HTS (Lackovic et al., 2010), displayed a competitive inhibition of both *Ld*GDP-MP and *h*GDP-MP with *K*_*i*_ values at 62 and 20 μM, respectively, reflecting a lower affinity for the leishmanial enzyme compared to the human counterpart.

A docking study of the identified competitive inhibitors on GDP-MP structural models showed that compound **A** binds to both *Ld*GDP-MP and *h*GDP-MP with similar potency and binding modes: the quinoline, piperazine, and *tert*-butyl groups occupying the same position as the GDP-mannose nucleotide, ribose and mannose moieties, respectively, in both catalytic sites (Daligaux et al., 2016a; Figure 1C). In contrast, compound **B** was found to bind more strongly to *Ld*GDP-MP compared to *h*GDP-MP, with the diisopropylphosphonate group located more deeply in the leishmanial enzyme catalytic pocket compared to the human one (Mao et al., 2017; Figure 1C). These *in silico* data are in agreement with the non-selective inhibition of both leishmanial and human GDP-MP by compound **A** and the specific competitive inhibition observed with compound **B** on *Ld*GDP-MP.

## Cellular *in vitro* Antileishmanial Activity and Cytotoxicity of Compounds A and B

Both compounds have been evaluated on *L. donovani* and *L. mexicana* axenic and intramacrophage amastigotes in two host cell models: the RAW264.7 macrophage cell line and primary Bone Marrow Derived Macrophages (BMDM; Mao et al., 2017). Compound **A** showed a moderate antileishmanial activity on both *L. mexicana* and *L. donovani* with IC_50_ values between 30 and 50 μM and between 12 and 28 μM on axenic and intramacrophage amastigotes, respectively (Mao et al., 2017; Table 1). These data are in agreement with the IC_50_ previously reported at 21.9 μM on *L. major* intramacrophage amastigotes by Lackovic et al. (2010). Moreover, this GDP-MP inhibitor showed some cytotoxicity on both RAW264.7 and BMDM macrophages, giving a low Selectivity Index (SI) in both host cell models. On the other hand, compound **B** exhibited a very interesting activity on *L. donovani* axenic amastigotes with an IC_50_ at the micromolar range (Mao et al., 2017; Table 1). However, it was inactive on *L. mexicana* axenic amastigotes, in line with the data obtained on the purified enzyme showing a specific competitive inhibition of *Ld*GDP-MP. In *L. donovani* intramacrophage amastigotes, the activity of compound **B** was maintained with an IC_50_ at the micromolar range in both host cell models (Mao et al., 2017; Table 1). Interestingly, this compound was also active on *L. mexicana* intramacrophage amastigotes with IC_50_ values at 1.5 and 8.6 μM on RAW264.7 and BMDM cell models, respectively, suggesting that an additional mechanism of action, distinct from the parasite GDP-MP inhibition, may be involved. Moreover, no cytotoxicity was observed with compound **B** on BMDM, giving a promising SI above 94 and 12 in *L. donovani* and *L. mexicana*, respectively (Mao et al., 2017; Table 1). Nevertheless, some cytotoxicity was observed on RAW264.7 macrophages, giving a low SI on this cell model. These differences could be due to distinct mechanisms of drug uptake and accumulation between host cell models, the BMDM being closer to physiological and clinical conditions as they are primary macrophages.

**Table 1:** Antileishmanial and cytotoxic activities of compounds **A** and **B**.

**Compounds**	**IC**_****50****_ **(μM)** **±** **SD**						**Cytotoxicity CC**_****50****_ **(μM)** **±** **SD**		**Selectivity index (CC**_****50****_**/IC**_****50****_**)**			
	***L. donovani***			***L. mexicana***					***L. donovani***		***L. mexicana***	
	**axenic amastigotes**	**infected RAW.264.7 macrophages**	**infected BMDM^b^**	**axenic amastigotes**	**infected RAW.264.7 macrophages**	**infected BMDM^b^**	**RAW264.7 macrophages**	**BMDM^b^**	**RAW264.7 macrophages**	**BMDM^b^**	**RAW264.7 macrophages**	**BMDM^b^**
**A**	30.68 ± 6.62	19.52 ± 4.53	12.18 ± 4.74	49.25 ± 0.26	27.52 ± 2.23	12.05 ± 1.27	62.06 ± 7.39	51.36 ± 3.45	3.2	4.2	2.3	4.3
**B**	1.06 ± 0.10	0.63 ± 0.14	1.06 ± 0.41	>100	1.49 ± 0.26	8.59 ± 2.44	1.53 ± 0.17	>100	2.4	>94.3	1.0	>11.6
Miltefosine^a^	2.08 ± 0.24	1.83 ± 0.22	0.83 ± 0.12	23.74 ± 2.81	52.62 ± 4.98	51.39 ± 5.34	>25	>25	13.7	>30.1	> 0.5	>0.5

*The table was adapted from Mao et al. (2017). The results expressed correspond to the mean of three independent experiments (± SD)*.

(a) *Miltefosine: reference compound*.

(b) *BMDM, Bone Marrow Derived Macrophages*.

## Conclusion and Future Directions

The mannose activation enzyme systems leading to GDP-mannose biosynthesis are essential for host-parasite interactions. Thus, GDP-MP, but also PMI and PMM, are interesting targets to be inhibited for impairing glycoconjugate biosynthesis. In this review, we focus on GDP-MP, this enzyme being responsible for GDP-mannose biosynthesis. GDP-MP is a druggable protein involved in the host-cell/parasite interactions, that has now been biologically and pharmacologically validated. Although ubiquitous, molecular modeling on both leishmanial and human GDP-MPs strongly suggests that specific inhibitors could be designed. From a rational design of 100 compounds based on leishmanial and human GDP-MP tertiary structural models, compound **B** appeared to be the most promising. In comparison with compound **A** which displayed a competitive inhibition of both leishmanial and human GDP-MP with moderate antileishmanial activities and a low SI, compound **B** showed a specific competitive inhibition of *Ld*GDP-MP and an activity on both *L. donovani* and *L. mexicana* intramacrophage amastigotes at the micromolar range giving an interesting SI above 10 in the BMDM host cell model. Therefore, the *in vivo* antileishmanial activity of this compound should be analyzed in order to determine its potency for the treatment of leishmaniasis. Further investigations will address *in vivo* antileishmanial evaluation, pharmacokinetics, and pharmacodynamics of compound **B** to confirm its status as a hit. Furthermore, the pathways altered in the parasite by compound **B** could be investigated in future works through glycomics analysis in order to study the impact of this inhibitor on the membrane glycoconjugate composition. Pharmacomodulations of compound **B** would also allow to optimize its selectivity and affinity for the target in *Leishmania*. However, the large molecular volume of this compound required to fill GDP-MP catalytic pocket (Mao et al., 2017), as well as its high polarity, could present challenges for downstream optimization. In order to assess the relative importance of GDP-MP in the most pathogenic leishmanial species, comparative functional analyses should be performed to optimize the inhibitor strategy.

In conclusion, compound **B** can be considered as an original and interesting hit to be optimized proving that GDP-MP inhibition is a promising strategy to impair host-parasite interactions. However, the capacity of this specific metabolism alteration to prevent drug resistance emergence is still to be proved.

## Author Contributions

SP wrote the manuscript. WM, TH-D, CC, and PL contributed to manuscript revision and read and approved the submitted version.

### Conflict of Interest Statement

The authors declare that the research was conducted in the absence of any commercial or financial relationships that could be construed as a potential conflict of interest.
